# Admixture mapping of pelvic organ prolapse in African Americans from the Women’s Health Initiative Hormone Therapy trial

**DOI:** 10.1371/journal.pone.0178839

**Published:** 2017-06-05

**Authors:** Ayush Giri, Katherine E. Hartmann, Melinda C. Aldrich, Renee M. Ward, Jennifer M. Wu, Amy J. Park, Mariaelisa Graff, Lihong Qi, Rami Nassir, Robert B. Wallace, Mary J. O'Sullivan, Kari E. North, Digna R. Velez Edwards, Todd L. Edwards

**Affiliations:** 1 Vanderbilt Epidemiology Center, Institute for Medicine and Public Health, Vanderbilt University Medical Center, Nashville, Tennessee, United States of America; 2 Division of Epidemiology, Department of Medicine, Vanderbilt University Medical Center, Nashville, Tennessee, United States of America; 3 Vanderbilt Genetics Institute, Vanderbilt University Medical Center, Nashville, Tennessee, United States of America; 4 Department of Obstetrics and Gynecology, Vanderbilt University Medical Center, Nashville, Tennessee, United States of America; 5 Department of Thoracic Surgery, Vanderbilt University Medical Center, Nashville, Tennessee, United States of America; 6 Department of Obstetrics and Gynecology, University of North Carolina, Chapel Hill, North Carolina, United States of America; 7 Department of Obstetrics and Gynecology, Georgetown University School of Medicine, Washington, District of Columbia, United States of America; 8 Department of Epidemiology, Gillings School of Global Public health, University of North Carolina, Chapel Hill, North Carolina, United States of America; 9 Division of Biostatistics, Department of Public Health Sciences, School of Medicine, University of California, Davis, Davis, California, United States of America; 10 Department of Biochemistry and Molecular Medicine, University of California, Davis, Davis, California, United States of America; 11 Department of Internal Medicine, University of California, Davis, Davis, California, United States of America; 12 Department of Epidemiology, University of Iowa College of Public Health, Iowa City, Iowa, United States of America; 13 Department of Obstetrics and Gynecology, Miller School of Medicine, Miami, Florida, United States of America; The Cleveland Clinic, UNITED STATES

## Abstract

Evidence suggests European American (EA) women have two- to five-fold increased odds of having pelvic organ prolapse (POP) when compared with African American (AA) women. However, the role of genetic ancestry in relation to POP risk is not clear. Here we evaluate the association between genetic ancestry and POP in AA women from the Women’s Health Initiative Hormone Therapy trial. Women with grade 1 or higher classification, and grade 2 or higher classification for uterine prolapse, cystocele or rectocele at baseline or during follow-up were considered to have any POP (N = 805) and moderate/severe POP (N = 156), respectively. Women with at least two pelvic exams with no indication for POP served as controls (N = 344). We performed case-only, and case-control admixture-mapping analyses using multiple logistic regression while adjusting for age, BMI, parity and global ancestry. We evaluated the association between global ancestry and POP using multiple logistic regression. European ancestry at the individual level was not associated with POP risk. Case-only and case-control local ancestry analyses identified two ancestry-specific loci that may be associated with POP. One locus (Chromosome 15q26.2) achieved empirically-estimated statistical significance and was associated with decreased POP odds (considering grade ≥2 POP) with each unit increase in European ancestry (OR: 0.35; 95% CI: 0.30, 0.57; p-value = 1.48x10^-5^). This region includes *RGMA*, a potent regulator of the BMP family of genes. The second locus (Chromosome 1q42.1-q42.3) was associated with increased POP odds with each unit increase in European ancestry (Odds ratio [OR]: 1.69; 95% confidence interval [CI]: 1.28, 2.22; p-value = 1.93x10^-4^). Although this region did not reach statistical significance after considering multiple comparisons, it includes potentially relevant genes including *TBCE*, and *ACTA1*. Unique non-overlapping European and African ancestry-specific susceptibility loci may be associated with increased POP risk.

## Introduction

Pelvic organ prolapse is characterized by the descent of pelvic organs including uterus, bladder and rectum into the vaginal space due to loss of underlying support system that normally holds these organs in their anatomic locations. POP is a common condition with up to 40% of women having some degree of prolapse after menopause. While not all POP requires surgical intervention or is symptomatic, it is one of the most common indications for gynecologic surgery in the US after uterine fibroids and endometriosis [1]. Estimates of lifetime risk of undergoing surgery for POP range from 11% to 19% [2; 3]. Factors such as aging, family history of POP, genetic predisposition, increasing parity, and higher body mass index (BMI) have been associated with greater risk for POP [4–6].

Additionally, race/ethnic status has been postulated to be associated with POP, with European Americans (EA) having 2–5 fold increased risk for POP than African Americans (AA) [7–11]. However, it is not clear if this disparity is due to biological/genetic differences or factors such as varying access to medical care and varying care-seeking behaviors between races/ethnicities. Anatomic comparative studies suggest AA women to have greater pelvic muscle mass and strength, a smaller angle in the pelvic arch/inlet due to closer attachment of the puborectalis muscle and increased pelvic floor mobility after vaginal delivery, than in EA women, providing potential reasons for elevated risk in EA women [12–14]. These evidence together suggest a potential role for genetic differences attributed to differences in continental ancestry in influencing POP risk.

Baseline evaluation of POP risk from the Women’s Health Initiative Hormone Therapy (WHI-HT) trial showed that AA women had 0.63 (95% confidence interval [CI]: 0.50, 0.79), 0.50 (95% CI: 0.44, 0.58) and 0.65 (0.59, 0.73) lower odds of having uterine prolapse, rectocele and cystocele, respectively, compared with EA women [7]. The WHI-HT is the largest multi-ethnic study conducted to-date with uniform assessment of objectively measured POP at baseline for all study participants and likely provides a relatively less biased assessment of the reported disparity compared with other studies. Additionally, the availability of genome wide association study (GWAS) data for AA women primes the WHI-HT as a unique resource to investigate the role of genetic ancestry in relation to POP risk. To our knowledge, this is the only existing resource available that provides information on POP and genome-wide data for African Americans.

Recently, we conducted a GWAS of POP risk in AA and Hispanic women from the WHI-HT, an analysis that was designed to detect common variants with similar allele frequencies in these two populations [15]. Here, we designed a study with the intention of investigating highly differentiated markers (between African and European populations) to delineate the role of local genetic ancestry in relation to POP risk in AA women from the WHI-HT. Admixture mapping is a method that complements traditional GWAS approaches and is a potentially powerful method of identifying causal genetic loci for diseases that vary in prevalence across populations. Admixture mapping differs from the traditional GWAS approach in the following ways. The method is most optimized to detect regions of disease susceptibility which are differentially distributed across ancestral populations and well represented in admixed populations such as African Americans who have genetic contributions from African and European subcontinents. It additionally derives greater power in detecting associations due to reduced multiple testing burden from the fewer number of markers that are required to cover the whole genome owing to long range ancestral linkage disequilibrium [16;17]. Leveraging genetic ancestry to detect the determinants of POP risk, the objectives of this study are to: 1) evaluate whether one or more genetic segments originating from African or European sub-continents (local ancestry) are associated with pelvic organ prolapse and 2) evaluate whether the average of the local ancestry estimates across an individual shows excess of European or African ancestry in relation to POP in a population of AA women from the WHI-HT.

## Materials and methods

### Study population

The WHI-HT trial is one arm of the WHI study, which overall enrolled over 160,000 post-menopausal women between 50–79 years of age from 40 clinical centers across the US from the years 1993 through 1998 to one of four clinical trials or the observational study [18]. Details regarding the WHI and the WHI-HT have been described elsewhere [18–20]. The WHI-HT is a large multi-ethnic clinical trial which enrolled 27,342 post-menopausal women of whom 2,739 self-identified as AA women, the target population for this analysis. De-identified data were accessed through authorized and secure methods with approval from the Institutional Review Board at Vanderbilt University, the Women's Health Initiative, and the database of Genotypes and Phenotypes (dbGaP).

### POP measurement: Case and control definitions

As a part of standard baseline procedure, all women participating in the WHI-HT received a pelvic exam conducted by a WHI-trained gynecologist, nurse or physician’s assistant. Using centrally validated and uniform procedures, trained WHI-staff evaluated the presence of uterine prolapse, cystocele and/or rectocele in a supine lithotomy position with or without the Valsalva maneuver. Presence and severity of POP was determined using the WHI POP classification system, where no prolapse was classified as grade 0, prolapse into the vagina was classified as grade 1, prolapse to the introitus was classified as grade 2 and prolapse beyond the introitus/hymen was classified as grade 3. In addition to baseline assessment, women underwent similar pelvic exams in one to ten yearly follow-up visits. For this sub-study women with grade 1 or higher uterine prolapse, rectocele and/or cystocele occurring either at baseline or at subsequent follow-up visits were considered to have any POP. To assess clinically significant POP, women were considered to have moderate/severe POP if they had uterine prolapse, rectocele and/or cystocele of grade 2 or higher at baseline or at any of the subsequent follow-up visits. Women were considered to be controls if they had grade 0 POP for all three types of prolapse: uterine prolapse, rectocele and cystocele in a minimum of two WHI visits including the baseline visit, and had no mention of POP from any other visits.

### Measurement of covariates

At baseline, WHI collected information on several demographic characteristics and medical history through standardized questionnaires, including age, self-identified race/ethnicity, reproductive history, and hysterectomy status. Anthropometric traits such as weight (kilograms) and height (cm) were measured at baseline and at subsequent follow-up visits. In addition to age at baseline, we constructed an age variable to reflect the age at first ascertainment within WHI visits for POP cases and the age at last visit for women who had no POP prior to being lost-to-follow-up or study completion, whichever came first, for controls. We used the participants’ BMI for the visit which corresponded to first ascertainment of POP at the WHI for cases and last visit prior to being lost-to-follow-up for controls.

### Genotyping and quality control (QC)

The SNP Health Association Resources (SHARe) is a study nested within the WHI and funded by the National Health Lung and Blood Institute (NHLBI) to evaluate genetic determinants of disease in approximately 8,420 AA and 3,587 Hispanic women who participated either in the WHI-HT study or the WHI observational study. These samples were genotyped with the Affymetrix Human SNP Array 6.0 (Affymetrix®, Inc Santa Clara, CA). Standard QC procedures were performed using PLINK and are detailed in S1 Text and S1 Fig [21].

Upon QC completion, there were 344 control participants for whom two or more pelvic exams were available to confirm absence of uterine prolapse, cystocele and rectocele. There were 805 AA women who had prolapse of any severity for any of the three types of prolapse either at baseline or follow-up visits; 156 of these were moderate/severe POP cases.

### Ancestry estimation

We used the software Local Ancestry in admixed Populations Ancestry (LAMP-ANC) with proxy ancestral allele frequency (Phase 3 1000 Genomes reference panels) [22] inputs for SNPs for Europeans and Africans to infer local ancestry across the genome for a total of 777,060 markers available in our post-QC dataset [23]. Local ancestry inference was performed using the following assumptions based on uncorrelated markers, seven generations since admixture began, recombination rate = 1x10^-8^, and average African and European admixture estimates of 0.8 and 0.2, respectively. Additionally, proportion of overlap between windows of ancestry inference was set to 0.2 and the r-squared threshold for LD-pruning was set to 0.1. Local ancestry was then coded as the number of European alleles at each marker (0, 1 or 2 European ancestry calls per marker). We then estimated the proportion of European ancestry for each individual (global ancestry) by summing the number of local European ancestry calls across all markers per person and then dividing by the total number of markers per person. Since the power of an admixture mapping study depends on ancestry informative markers, we limited our evaluation of ancestry calls to markers that were found to have an absolute difference in allele frequency, Δ of ≥0.4 between EUR and AFR populations in the 1000 Genomes. In the present dataset, 39,546 markers had a Δ of 0.4 or higher and were utilized for testing the association between local ancestry and POP.

### Statistical analyses

We used multiple logistic regression using StataIC, version 12 (StataCorp, College Station, TX, USA) to evaluate the association between global ancestry (% European ancestry) and POP (any POP and moderate/severe POP), adjusting for age at ascertainment (continuous), parity (continuous) and body mass index (BMI, continuous). The associations between local ancestry and POP (any POP and moderate/severe POP) were tested using two methods: 1) case-only and 2) case-control. Briefly, a case-only design compares the deviation in the frequency of estimated ancestry at each marker compared with the genome-wide average in cases, which makes it a highly sensitive test, but may also be prone to detecting false-positive signals in the absence of a proper comparator group and due to its inability to adjust for other correlated factors [16]. We computed a Z-statistic ((Local ancestry frequency at marker-_i_−global ancestry frequency)/standard error of local ancestry frequency at marker-_i_) for each locus and calculated two-sided p-values. We conducted the case-control admixture mapping analyses with logistic regression using PLINK, where we regressed POP (any POP and moderate/severe POP) onto local ancestry, adjusting for aforementioned variables and two multi-dimensional scaling (MDS) components representing continuous axes of genetic ancestry. The motivation behind conducting two different types of tests (case-only and case-control study) was based on the intuition that overlapping signals from both tests in a given region provides greater likelihood of identifying a true positive, while weeding out false-positive signals. Therefore, regions from admixture mapping analyses which showed overlaps between case-only and case-control methods with suggestive peak p-values (<1x10^-3^), within two integers on the–log_10_p-value scale to the left and right of the strongest case-control peak for a given region were warranted further attention. Statistical significance for admixture mapping analyses was established empirically with 10,000 case-control permutation tests at p-value = 1.82x10^-5^, implying approximately 2,747 independent ancestry markers.

Significant or suggestive peaks from admixture mapping analyses were further assessed for evidence of single SNP association with POP using genotyped and imputed variants as secondary analyses. Genotype data for broad regions (10 to 20 Mb regions) below suggestive/significant peaks were imputed using the 1000 genomes cosmopolitan reference panels using IMPUTE2 [24]. Association between SNPs and POP (any POP and moderate/severe POP) were performed using multiple logistic regression adjusting for age at ascertainment, BMI, parity and the two MDS components to represent continuous axes of genetic ancestry using SNPTEST [25]. Then, to evaluate if any of the SNPs investigated in the regions of interest explained the admixture mapping peaks, we performed logistic regression between local ancestry and POP conditioning on the most-statistically significant SNPs contained within the region of interest.

## Results

Women with any POP and moderate/severe POP were more likely to have higher parity on average, compared with controls. At the WHI baseline visit, women without POP were slightly younger (mean age: 60.1) than women with any POP (mean age: 61.8) and women with moderate/severe POP (mean age: 62.8) (Table 1). However, controls were more likely to be older when considering age at ascertainment than cases since women were only considered controls in this sub-study because we recorded their age at last visit without POP prior to being lost to follow up or study’s end. Of the 805 any POP cases, 292 women developed POP during follow-up visits. Similarly, of the 156 women who had moderate/severe POP in our study, 98 women developed moderate/severe POP during follow-up visits.

**Table 1:** Characteristics of African American POP cases and controls.

Variable	Controls (N = 344)	Any POP (N = 805)	Grade 2–3 POP (N = 156)
	**Mean (SD)**	**Mean (SD)**	**Mean (SD)**
Age at baseline	60.11 (6.68)	61.77 (6.96)	62.79 (6.77)
Age at ascertainment	65.20 (6.80)	62.75 (7.02)	64.85 (6.98)
Body mass index (kg/m^2^)	31.19 (6.59)	31.71 (6.19)	31.71 (6.07)
Parity (# child births)	2.26 (1.59)	2.85 (1.64)	3.28 (1.60)
Hysterectomy at baseline (%)	23.20%	42.20%	34.80%
European Genetic Ancestry (%)	24.30%	23.15%	23.05%
**POP Type**	**-**	**N (%)** ^**a**^	**N (%)** ^**a**^
Cystocele	-	737 (91.5%)	134 (85.9%)
Rectocele	-	372 (46.2%)	46 (29.5%)
Uterine Prolapse	-	189 (23.5%)	10 (6.4%)

POP: pelvic organ prolapse.

a Numbers do not add up to total N and percentages do not add up to 100% as these are not mutually exclusive conditions; cases may have one or more type of prolapse.

**Fig 1:**
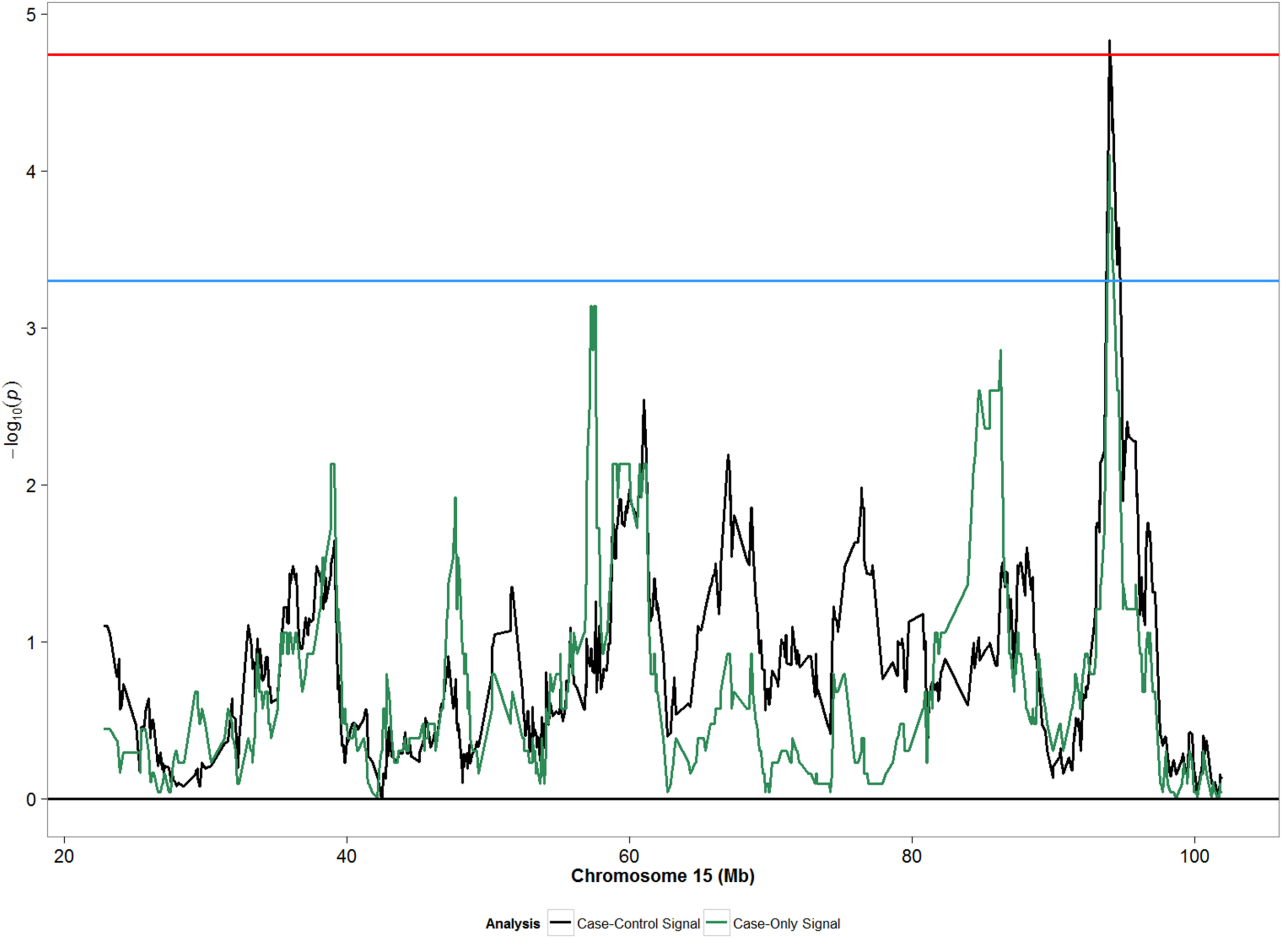
Admixture mapping peaks for chromosome 15 from case-only and case-control designs considering moderate/severe POP (grades 2–3) in African Americans. Blue horizontal line: suggestive p-value threshold; Red horizontal line: permutation based p-value threshold.

**Fig 2:**
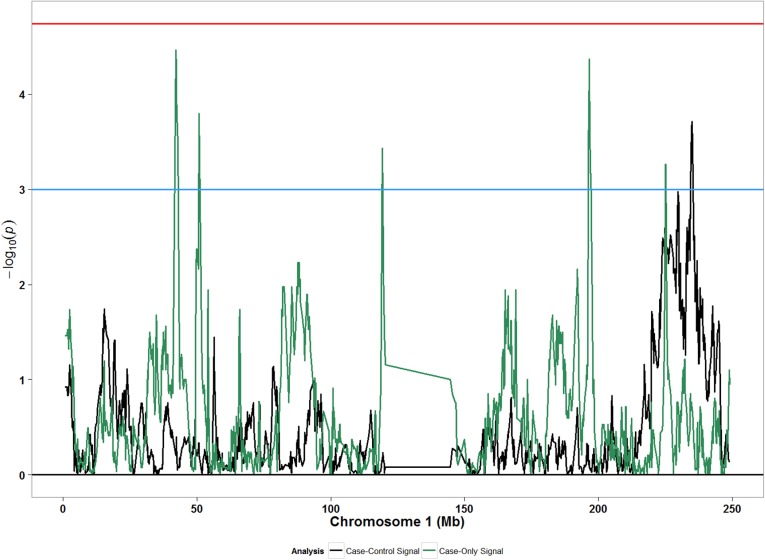
Admixture mapping peaks for chromosome 1 from case-only and case-control designs considering any POP (grades 1–3) in African Americans. Blue horizontal line: suggestive p-value threshold; Red horizontal line: permutation based p-value threshold.

We did not observe any meaningful associations between global ancestry and POP (Table 2). Compared with African ancestry, adjusted odds ratio (OR) for European ancestry was 0.82 (95% CI: 0.31, 2.16) considering any POP; similar effect sizes were observed for moderate/severe POP. In analyses assessing local ancestry, both case-only and case-control approaches detected statistically significant associations with local ancestry at chromosome 15 (90 to 100 mega-bases [Mb]) for moderate/severe POP (Fig 1), and suggestive signals at chromosome 1 (220–240 Mb) for any POP (Fig 2). The strongest admixture mapping signal in case-control analyses was observed in the chromosome 15q26.3 region, where each unit increase in European ancestry was associated with decreased odds of moderate/severe POP (OR: 0.35; 95% CI: 0.23, 0.57; p: 1.48x10^-5^) (Table 3). Evaluation of the same association in any POP showed an attenuated association that was yet in the same direction as the association from moderate/severe POP analysis (Table 3). Sensitivity analyses of our main finding for the top ancestry locus separating incident and prevalent cases showed greatest agreement with incident cases for moderate/severe POP (S1 Table).

**Table 2:** Association between European-ancestry percent in relation to POP in African American women.

Model	N-Controls/N-Cases	OR	(95% CI)	P
Grade 0 vs. Grade 1–3 POP	341/794			
European Ancestry		0.82	(0.31, 2.16)	0.71
Grade 0 vs Grade 2–3 POP	341/155			
European Ancestry		0.81	(0.19, 3.39)	0.77

OR = odds ratio; CI = confidence interval; Models were adjusted for age at ascertainment, body mass index and parity.

**Table 3:** Associations between local European ancestry and POP in top regions.

Region	Nearby Genes	Classification	Ancestry OR (95% CI)	P_case-control_	P_case-only_^c^
15q26.2	RGMA, CHD2	Grade 0 vs. 2/3	0.35 (0.22, 0.57) ^a^	1.48x10^-5^	7.95x10^-5^
		Grade 0 vs. 1–3	0.77 (0.59, 0.99) b	0.049	-
1q42.1–42.3	ARID4B, TBCE, ACTN2, PGBD5, ACTA1	Grade 0 vs. 1–3	1.69 (1.28, 2.22) b	1.93x10^-4^	6.7x10-4
		Grade 0 vs. 2/3	1.86 (1.26, 2.76) ^a^	2.00x10^-3^	-

OR: Odds Ratio; CI: Confidence Interval.

a Modeled against local European ancestry adjusted for covariates (age at ascertainment, BMI, parity and continuous axes of MDS components)

b Modeled against local European ancestry adjusted for covariates (age at ascertainment, BMI, parity and continuous axes of MDS components)

c Case-only p-values were used along with case-control p-values to find overlapping regions.

**Fig 3:**
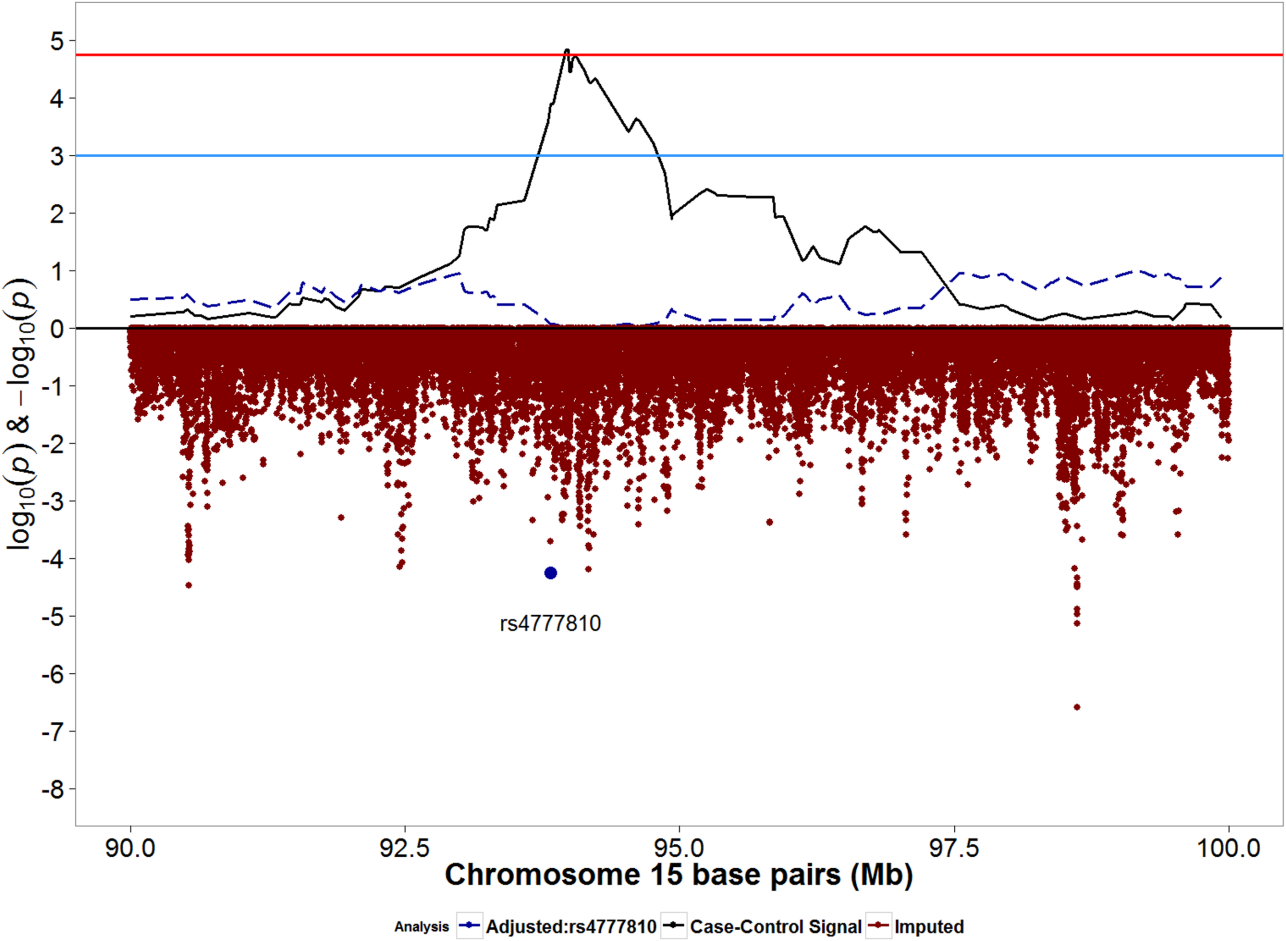
Signals from moderate/severe case-control admixture mapping and imputed SNPs for chromosome 15q26.2 region. Blue horizontal line: suggestive p-value threshold; Red horizontal line: permutation based p-value threshold. Solid black line represents admixture mapping signal prior to conditional analysis. Red dots represents log10(p-values) for genotyped and imputed SNPs within the admixture mapping peak. Blue dot represents SNP rs4777810, the most significant SNP directly below the admixture mapping peak. Dashed blue line represents admixture mapping signal after adjustment for SNP rs4777810.

We then evaluated the association between imputed SNPs in the region below the admixture mapping peak to identify potential SNPs which may be associated with POP. Admixture mapping peaks (-log_10_(p-values)) from case-control analyses and SNP associations (log_10_(p-values)) are juxtaposed in Fig 3 for chromosome 15. Imputed SNP rs4777810 was the most statistically significant SNP under the peak (Fig 3; Table 4). Compared with the reference allele (A), the effect allele (G) was associated with decreased POP risk (OR: 0.37; 95% CI: 0.23, 0.50; p: 5.58x10^-5^). Additional adjustment for rs4777810 severely attenuated the admixture mapping signal (Fig 3) and decreased the magnitude of ancestral odds ratio from 0.35 to 0.50) (Table 5). The effect allele for rs4777810 (risk decreasing allele) is found in higher frequency in the European reference population (57%) than in the African reference population (3%).

**Table 4:** Associations between genetic markers and POP in top regions identified from admixture mapping.

Classification	SNP	Region	BP	SNP OR (95% CI)	P	EA/RA	EAF	EAF- YRI/ASW/CEU
Grade 0 vs. 2/3	rs4777810	15q26.2	93825164	0.37 (0.23, 0.50) ^a^	5.58x10^-5^	G/A	0.19	0.03/0.17/0.57
							
Grade 0 vs. 1–3	rs78992478	1q42.1–42.3	235397083	3.15 (1.93, 5.14) ^b^	4.23x10^-6^	C/T	0.92	0.98/0.94/1.00
	rs2501094	1q42.1–42.3	225095329	1.63 (1.32, 1.64) ^b^	5.47x10^-6^	C/A	0.55	0.50/0.63/0.99

SNP: Single Nucleotide Polymorphism; BP: Base Pair; P: P-value; OR: Odds Ratio; CI: Confidence Interval; EA: Effect Allele; RA: Reference Allele; EAF: Effect Allele Frequency.

a Modeled against SNP, adjusted for covariates (age at ascertainment, BMI, parity and continuous axes of MDS components).

b Modeled against SNP, adjusted for covariates (age at ascertainment, BMI, parity and continuous axes of MDS components).

**Table 5:** Associations between local ancestry and POP with and without adjustment for genetic markers in top regions.

Classification	Region	Adjusted for SNP	Ancestry OR (95% CI) ^a^	P
Grade 0 vs. 2/3	15q26.2	No	0.35 (0.22, 0.57)	1.48x10-5
		rs4777810	0.50 (0.30, 0.85)	0.01
Grade 0 vs. 2/3	1q42.1–42.3	No	1.69 (1.28, 2.22)	1.93x10-4
		rs78992478	1.56 (1.18, 2.06)	1.75x10-3
		rs2501094	1.48 (1.11, 1.97)	6.80x10-3
		rs78992478 + rs2501094	1.37 (1.03, 1.32)	0.03

SNP: Single Nucleotide Polymorphism; OR: Odds Ratio; CI: Confidence Interval; P: P-value

a Modeled against local European ancestry adjusted for covariates (age at ascertainment, BMI, parity and continuous axes of MDS components), with and with and without adjustment for top imputed/genotyped marker(s) at region of interest.

**Fig 4:**
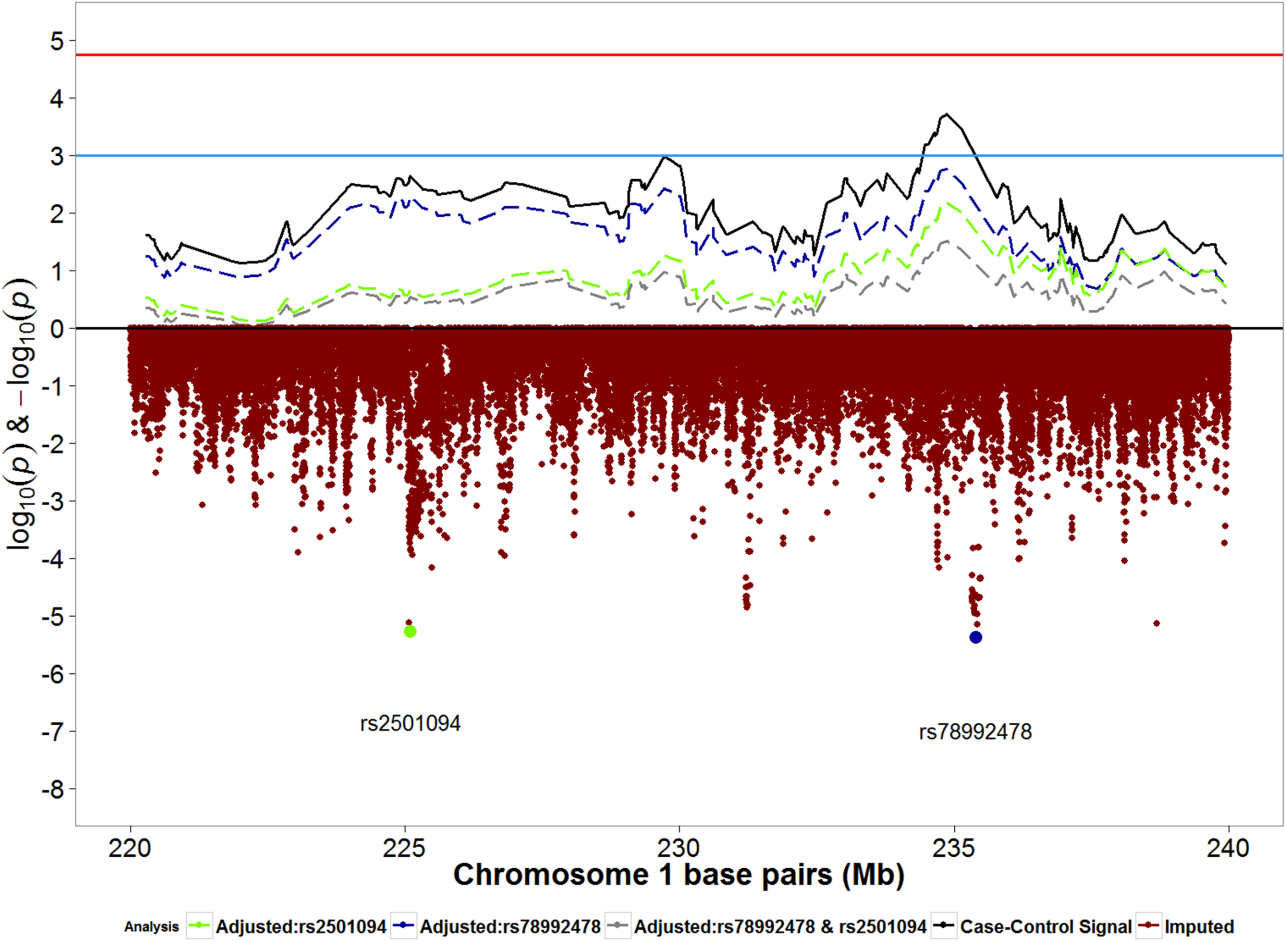
Signals from any-POP case-control admixture mapping and imputed SNPs for chromosome 1q42.1–42.3 region. Blue horizontal line: suggestive p-value threshold; Red horizontal line: permutation based p-value threshold. Solid black line represents admixture mapping signal prior to conditional analysis. Red dots represents log10(p-values) for genotyped and imputed SNPs within the admixture mapping peak. Blue dot represents SNP rs78992478, the most significant SNP directly below the admixture mapping peak. Green dot represents SNP rs2501094, the second-most significant SNP directly below the admixture mapping peak. Dashed blue line represents admixture mapping signal after adjustment for SNP rs78992478. Dashed green line represents admixture mapping signal after adjustment for SNP rs2501094. Dashed grey line represents admixture mapping signal after adjustment for both SNPs.

The second strongest admixture mapping signal from case-control analyses was observed in the chromosome 1q42.1–42.3 region, where each unit increase in European ancestry was associated with increased odds of any POP (OR: 1.69; 95% CI: 1.28, 1.22; p: 1.93x10^-4^) (Table 3). Evaluation of the same association in moderate/severe POP for comparison showed that the effect estimate was stronger in the moderate/severe POP analysis than in the any POP analysis, however with a larger p-value due to smaller sample size (Table 3). We then evaluated the association between imputed SNPs in the region below the admixture mapping peak in relation to any POP. In analyses of imputed SNPs, SNP rs78992478 was directly below the admixture mapping peak (Fig 4) and compared with the reference allele T, the effect allele C was associated with increased risk for any POP (OR: 3.15; 95% CI: 1.93, 5.14; p: 4.23x10^-6^) (Table 4).

The second most significant SNP in the admixture mapping peak in chromosome 1 was rs2501094, for which effect allele C was associated with increased risk for POP (OR: 1.63; 95% CI: 1.32, 2.02; p: 5.47x10^-6^) (Table 4 and Fig 4). In models evaluating the association between local ancestry and any POP, additionally adjusting for SNP rs78992478 and/or rs2501094 decreased the admixture mapping signal (Fig 4) and also decreased the odds ratio for ancestry from 1.69 to 1.37 (Table 5). The largest drop in admixture mapping signal was seen with adjustment for rs2501094 (Table 5). The effect allele for rs2501094 (risk increasing allele in this case) is found in higher frequency in the European reference population (99%) than in the African reference population (50%).

**Fig 5:**
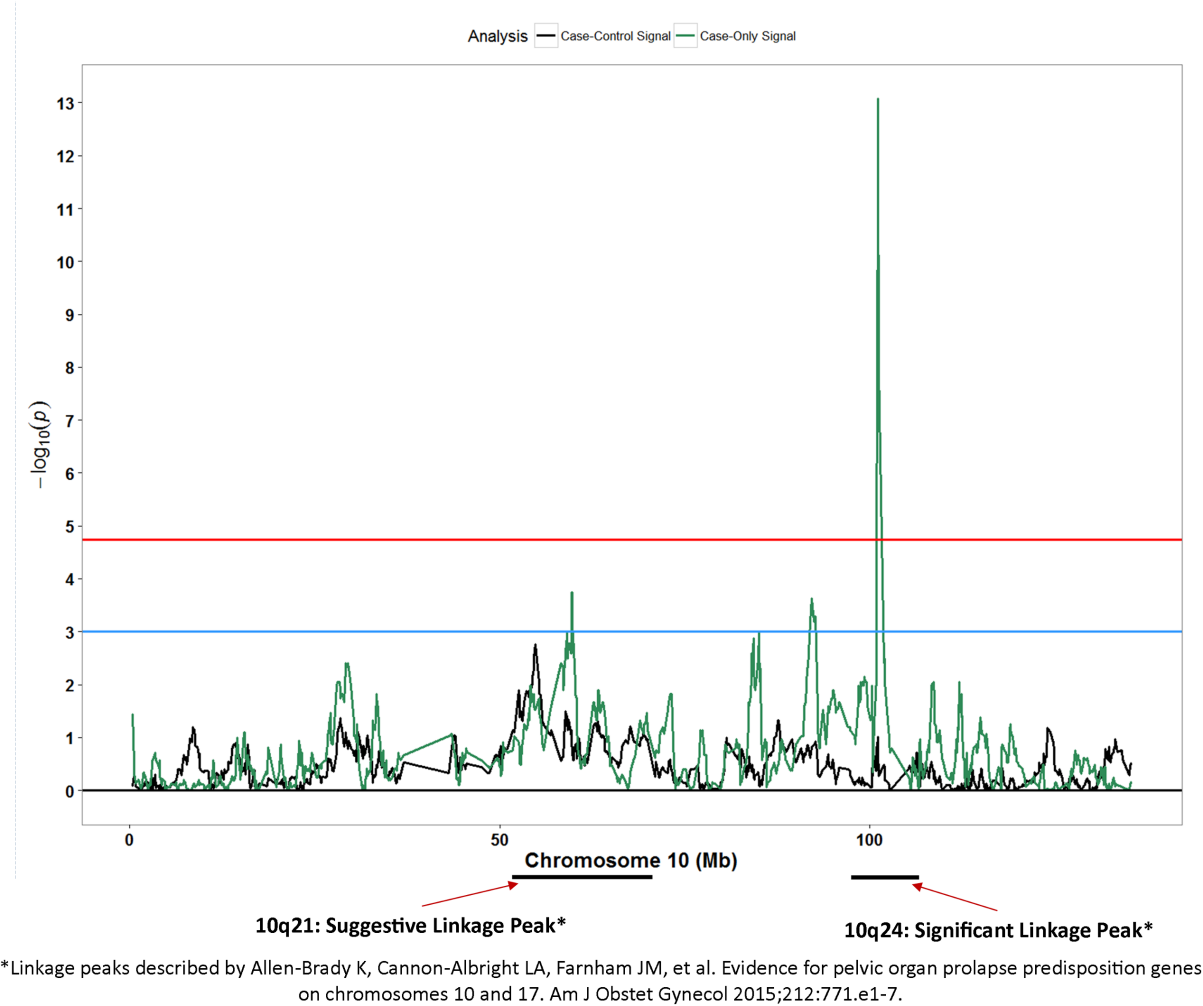
Admixture mapping peaks for chromosome 10 in context with previously reported linkage peak. Blue horizontal line: suggestive p-value threshold; Red horizontal line: permutation based p-value threshold; Black peaks: case-control admixture mapping; Green peaks: case-only admixture mapping.

Secondary to the results mentioned above, we compared results from our case-only and case-control admixture mapping signals with SNPs or regions reported in previously reported studies evaluating genetic regions with respect to POP. We observed patterns of association for case-only signals (Fig 5) in chromosome 10q21 and 10q24 (Z-score:-7.46; p: 8.5x10^-14^) regions that were strikingly similar to linkage peaks reported by Allen-Brady and colleagues [26].

## Discussion

As the first study to evaluate the association between genetic ancestry and POP, our results suggest presence of two unique ancestry-specific susceptibility loci, where in one region European ancestry was associated with increased risk for POP, and in another region African ancestry was associated with increased risk for POP. Contrary to epidemiological evidence that show of higher prevalence of POP in EAs than in AAs, our evaluation of genetically inferred European ancestry proportion per person was not associated with POP. There was not sufficient evidence in this study to support the idea that POP disparity between EA and AA is attributable to differences in genetic ancestry averaged over the entire genome. While EA women in the WHI were more likely to have POP at baseline than AA women, POP was common in both populations (40.2% in EA vs. 29.4% in AA) [7]. For such a highly prevalent complex condition, it is plausible that women from European or African ancestries may have shared susceptibility loci and there may be distinct ancestry-specific susceptibility loci which are associated with increased or decreased risk for POP, as has been shown for prostate cancer [27]. The presence of opposing effect-estimates in ancestry-specific loci could have potentially diluted the association between global ancestry and POP as it is merely a representation of average ancestry along the genome. Alternatively, if there is an excess of disease susceptibility loci in European Americans with meaningful but small allele frequency differences, these would have been missed in our study as we focused on variants with large differences; we were only powered to detect these. However, due to lack of availability of well characterized cohorts such as the WHI, we were not able to test these associations in an independent African American population for who data on POP and genetics are simultaneously available and should therefore be interpreted with care.

The chromosomal region 1q42.1-q42.3 showed a greater presence of excess European ancestry in POP cases than in controls. Although the signal for this region was not statistically significant (p: 1.93x10^-4^) after considering multiple comparisons (p-value threshold: 1.8x10^-5^), several pieces of other evidence collectively point to the plausibility that the signal from this region may be of interest in relation to POP. This 20 Mb region harbors several genes which may be related to maintenance of components of the pelvic support system and may be related to POP, including tubulin binding cofactor E (*TBCE*) and alpha-actin-1 (*ACTA1*) genes.

*TBCE* is a peripheral associated membrane protein which plays an essential role in polymerizing microtubules [28]. This gene has been suggested to play a major role in forming neuromuscular junctions [29] and mutations in the *TBCE* gene have shown to cause loss of microtubule formation in distal ends axons [30;31]. Denervation of major muscles involved in the pelvic support system due to stress-related insult during labor has been hypothesized as an important contributor to POP. It is plausible that altered expression of the *TBCE* gene may impact proper repair of denervation sites. The *ACTA1* gene product is a globular protein that is important in thin microfilament formation including F-actin and G-actin filaments and plays an essential role in muscular contraction [32]. A recent study that evaluated F-actin expression in vaginal fibroblasts reported relative F-actin expression was higher in fibroblasts from women with POP than from women without POP [33].

However, with such a broad peak of 20 MB, it is difficult to delineate whether the region is foretelling several signals for which the granularity has been lost or from one signal. When we adjusted for the most significant marker under the peak (rs78992478), it did not significantly change the effect estimate or the peak of the admixture mapping signal. This is likely due to a lack of correlation with the admixture mapping peak, which is also reflected by the relatively smaller allele frequency difference across populations for this SNP. However, adjusting for a SNP at the second most significant locus under the broad peak (approximately 10 Mb away—rs2501094), the admixture mapping signal attenuated slightly more, with the greatest attenuation when both SNPs were adjusted for. Intriguingly, our previous GWAS investigation of pelvic organ prolapse using African American and Hispanic women identified a common marker across those populations near gene *PGBD5* in the 1q42.1–3 region. This further suggests a more complicated genetic architecture for pelvic organ prolapse with the possibility that if genetic variants affect POP risk, there may be risk increasing variants that are common to and specific to populations.

The most persuasive case-control admixture mapping peak, which remained significant after permutation testing, is located at chromosome 15q26.2. Interestingly, European ancestry was inversely associated with moderate/severe POP. This region harbors the repulsive guidance molecule family member a (*RGMA*) gene, which is a glycosylphospatidylinositol-anchored glycoprotein. *RGMA* was initially discovered for its role as an axon guidance protein in the central nervous system [34;35]. The RGM family of genes including *RGMA* have been shown to be important regulators of the bone morphogenic protein (BMP) pathway including the *BMP-1* gene [36;37]. The *BMP-1* gene is involved in activation of the lysl oxidase (LOX) family of genes and plays a crucial role in maturation of procollagen chains and elastin [38]. A small study evaluating the association between POP cases and controls showed decreased expression of the *BMP-1* gene in POP cases compared with controls [39]. The SNP identified for this region (rs4777810) not only explained the admixture mapping peak suggesting a high degree of correlation with the peak, but also falls in a gene regulatory region as predicted by the Regulome database, with a regulatory score of 2b [40]. The SNP is located in a transcription factor binding region and is on a DNAse hypersensitivity region as detected by DNA foot printing assay and DNase peak identification. Specifically, the SNP lies in a region that acts as a binding site for the *EZH2* gene product, a histone–lysine N-methyltransferase enzyme that facilitates transcriptional repression. *EZH2* in turn has been shown to regulate *RGMA* expression [41].

Several candidate gene studies, two GWAS [15;42] and three genome-wide linkage studies have evaluated the association between genetic variants and POP with limited replication of associations across studies.[43] In the first GWAS for POP, Allen-Brady and colleagues identified six loci (4q21, 8q24, 9q22, 15q11, 20p13, and 21q22) with genome-wide statistical significance in a population of EA women [42]. The second GWAS, undertaken in AA and Hispanic women from the WHI-HT [15], reported several loci with suggestive statistical significance but found no evidence of association for previously reported loci. For complex polygenic diseases such as POP, it is possible that women with European and African ancestries may have shared and/or unique ancestry-specific susceptibility loci, especially considering the differences in POP prevalence between these populations. The most recent genome-wide linkage study by Allen-Brady and colleagues identified statistically significant linkage peaks in chromosomes 17q25 and 10q24-26 regions [26]. Post-hoc comparisons of case-only admixture mapping signals from our study to those present in the literature revealed a conspicuous resemblance in association peaks for the chromosome 10q24 region noted by Allen-Brady and colleagues [26]. A notable candidate gene in this region includes another gene from the LOX family of genes, LOX-like-4 (*LOXL-4*). Even though this finding is incidental, as it deviates from our *a priori* criteria of considering only overlapping signals from both case-only and case-control admixture mapping analyses, the striking resemblance with previously reported peaks is noteworthy.

We took measures at the design and analysis phase to ensure internal validity. We reduced misclassification of cases and controls, when possible, to identify controls at baseline who developed POP during follow-up. With the intention of reducing misclassification, even at the expense of losing controls from our previously utilized control definition [15], we limited our definition of controls to women with at least two measurements of POP at different time points. We additionally showed that the effect estimates for candidate local ancestry regions in relation to POP were in the same direction for moderate/severe POP and any POP (S2 Fig); estimates were stronger for moderate/severe POP at the top markers than for any POP, at the top regions (Table 3). To minimize false positives due to chance, that is especially likely in a case-only approach, or bias, we only considered overlapping regions from case-only and case-control designs with at least suggestive statistical significance. However, at the expense of minimizing false positives, our study may have missed potentially true signals such as the one observed for chromosome 10q24 in the case-only design. Since women without a uterus could still potentially have other forms of prolapse such as cystocele and rectocele we included these women in our study, with the understanding of potential selection bias especially if women with a hysterectomy were more likely to be controls. However, this concern is alleviated since similar signals were observed in case-only and case-control designs, the former of which would not be affected by hysterectomy status. To compensate for a small sample size in our study and to reduce multiple comparisons we limited our analysis to highly differentiated markers in the ancestral reference populations. Additionally, we used a rigorous method, permutation testing, to empirically estimate statistical significance from our primary admixture mapping analyses. In addition to identifying broad genetic regions from admixture mapping, we attempted to identify markers in the region that explained the admixture mapping peaks.

In conclusion, the results from our study suggest that POP is a genetically complex condition with susceptibility loci that may vary substantially in frequency between European and African ancestry populations. We provide evidence for two novel biologically plausible loci for POP risk and provide further evidence for a previously reported locus. Replication and fine-mapping studies in larger and similarly well-classified independent AA populations are a priority to confirm findings from this study.

## Supporting information

S1 Text Quality control details for genotype data. (DOCX)

S1 Fig QC flow chart for African American cases and controls from the WHI-HT trial. (PNG)

S2 Fig Comparing effect estimates of any POP and moderate/severe POP admixture mapping analyses for candidate admixture mapping regions. Top right quadrant contains effect estimates from local ancestry analyses in chromosome 1q42.3 region (orange dots) against any POP or moderate/severe POP. Bottom left quadrant contains effect estimates from local ancestry analyses in chromosome 15q26.2 region (blue dots) against any POP or moderate/severe POP.
(PNG)

S1 Table Admixture mapping sensitivity analyses for moderate/severe POP for the most significant marker chromosome15q23.1 region. Models adjusted for age, BMI, parity and average genetic ancestry. Incident cases refer to moderate/severe POP cases that developed during follow-up examinations. Prevalent cases refer to moderate/severe POP cases that were present at baseline examination. All cases refer to a combination of prevalent and incident moderate/severe POP cases. Stringent controls refer to individuals who had at least two WHI pelvic exams during baseline and follow-up and were confirmed to be absent for POP. Controls at baseline refer to individuals who did not have POP at baseline; these include individuals who did not develop POP during follow-up and also includes individuals who developed POP during follow-up.
(DOCX)
