# Supplementary material for: Admixture mapping of pelvic organ prolapse in African Americans from the Women’s Health Initiative Hormone Therapy trial
Source: PLoS One. 2017 Jun 5;12(6):e0178839. doi: 10.1371/journal.pone.0178839 (PMC5459562; doi:10.1371/journal.pone.0178839)
Supplement: S1 Text — (DOCX) [file pone.0178839.s001.docx]

**Supporting Information**

**S1 Text. Quality control details for genotype data**

Genotyping and Quality Control

The SNP Health Association Resources (SHARe) is a nested study within the WHI funded by the National Health Lung and Blood Institute (NHLBI) to evaluate genetic determinants of disease in approximately 12,007 AA and Hispanic women. These samples were genotyped with the Affymetrix Human SNP Array 6.0 (Affymetrix®, Inc Santa Clara, CA) whole genome genotyping platform, which includes a total of 934,940 oligos/SNPs. Of the 12,007 AA and Hispanic specimens, 8,420 specimens belonged to AA women who participated either in the WHI-HT study or the WHI observational study. We performed standard quality control (QC) procedures on these samples prior to analysis using PLINK (S1 Fig). Briefly, SNPs with minor allele frequencies less than 1% were first removed followed by individuals who had genotype call rates <95%. We then removed SNPs with a low genotyping rate (<95%) followed by individuals for whom X chromosome homozygosity rates for common SNPs (MAF ≥ 0.2) were ≥ 0.2. We then removed individuals who were found to have cryptic relatedness at the first-degree level of relatedness and for whom information on POP was not available either because they were not participants of the WHI-HT or because they did not have pelvic exams to confirm the presence or absence of POP. As mentioned above, we also removed controls for whom only one POP exam was available in the WHI-HT. We then removed SNPs which were found to violate Hardy-Weinberg equilibrium at the p-value threshold of 1x10^-6^. We finally removed SNPs which were not available in the 1000 genomes reference panels or for which the proper strand could not be identified.
