# Supplementary figures and images for: Admixture mapping of pelvic organ prolapse in African Americans from the Women’s Health Initiative Hormone Therapy trial
Source: PLoS One. 2017 Jun 5;12(6):e0178839. doi: 10.1371/journal.pone.0178839 (PMC5459562; doi:10.1371/journal.pone.0178839)

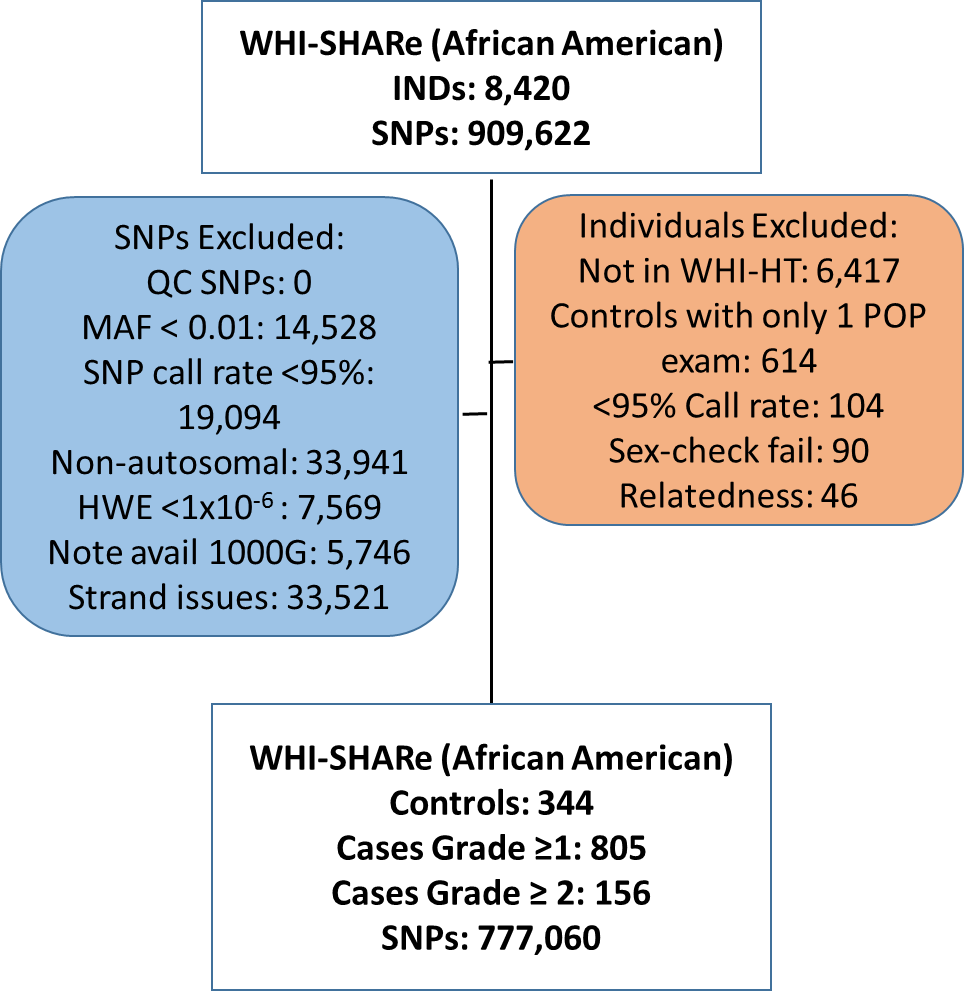

Supplement: S1 Fig — (PNG) [file pone.0178839.s002.png]

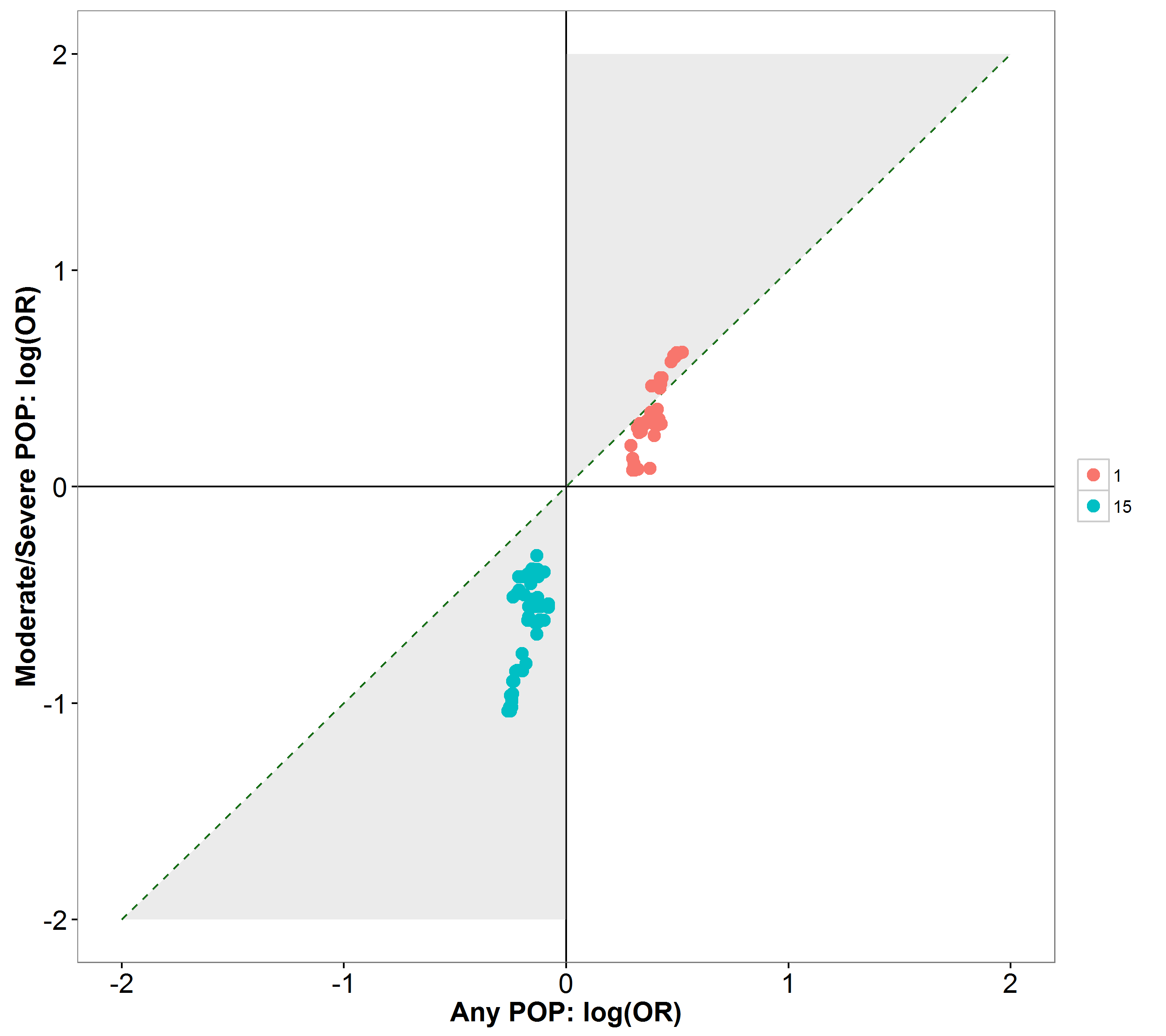

Supplement: S2 Fig — Top right quadrant contains effect estimates from local ancestry analyses in chromosome 1q42.3 region (orange dots) against any POP or moderate/severe POP. Bottom left quadrant contains effect estimates from local ancestry analyses in chromosome 15q26.2 region (blue dots) against any POP or moderate/severe POP. (PNG) [file pone.0178839.s003.png]
