# Supplementary material for: Admixture mapping of pelvic organ prolapse in African Americans from the Women’s Health Initiative Hormone Therapy trial
Source: PLoS One. 2017 Jun 5;12(6):e0178839. doi: 10.1371/journal.pone.0178839 (PMC5459562; doi:10.1371/journal.pone.0178839)
Supplement: S1 Table — Models adjusted for age, BMI, parity and average genetic ancestry. Incident cases refer to moderate/severe POP cases that developed during follow-up examinations. Prevalent cases refer to moderate/severe POP cases that were present at baseline examination. All cases refer to a combination of prevalent and incident moderate/severe POP cases. Stringent controls refer to individuals who had at least two WHI pelvic exams during baseline and follow-up and were confirmed to be absent for POP. Controls at baseline refer to individuals who did not have POP at baseline; these include individuals who did not develop POP during follow-up and also includes individuals who developed POP during follow-up. (DOCX) [file pone.0178839.s004.docx]

**S1 Table. Admixture mapping sensitivity analyses for moderate/severe POP for the most significant marker chromosome15q23.1 region**

| Analysis Type | N-Cases/N-Controls | OR | 95% CI | P |
| --- | --- | --- | --- | --- |
| 1. All Cases/Stringent Controls | 155/341 | 0.35 | (0.22, 0.57) | 1.48x10^-5^ |
| 1. Incident Cases/Stringent Controls | 97/341 | 0.31 | (0.18, 0.65) | 3.04x10^-4^ |
| 1. Prevalent Cases/Stringent Controls | 58/341 | 0.50 | (0.26, 1.00) | 4.45x10^-2^ |
| 1. Prevalent Cases/Controls at baseline | 58/1044 | 0.69 | (0.43, 1.11) | 1.80x10^-1^ |

Models adjusted for age, BMI, parity and average genetic ancestry. Incident cases refer to moderate/severe POP cases that developed during follow-up examinations. Prevalent cases refer to moderate/severe POP cases that were present at baseline examination. All cases refer to a combination of prevalent and incident moderate/severe POP cases. Stringent controls refer to individuals who had at least two WHI pelvic exams during baseline and follow-up and were confirmed to be absent for POP. Controls at baseline refer to individuals who did not have POP at baseline; these include individuals who did not develop POP during follow-up and also includes individuals who developed POP during follow-up.

Commentary for Table S1: These sensitivity analyses suggest results observed in our main analyses for moderate/severe POP are more reflective of incident POP cases. These analyses also caution the consequences of using only baseline data to determine case-control status especially when follow-up data on case determination is available. The effect of classifying would-be-cases during follow-up as controls at baseline can lead to a bias towards the null.
